# Supplementary material for: Pluripotency, Differentiation, and Reprogramming: A Gene Expression Dynamics Model with Epigenetic Feedback Regulation
Source: PLoS Comput Biol. 2015 Aug 26;11(8):e1004476. doi: 10.1371/journal.pcbi.1004476 (PMC4550282; doi:10.1371/journal.pcbi.1004476)
Supplement: S1 Text — (PDF) [file pcbi.1004476.s001.pdf]

Supplementary information about the five-gene model.

Similar to the four-gene model, depending on the dominance between the positive and negative feedback of gene x_1, the five-gene model showed three behaviors: (i) fixed-point attractor with high expression of pluripotent genes (FP), (ii) fixed-point attractor with high expression of differentiation genes (FD), and (iii) the oscillatory state (O). The five-gene model also showed differentiation from the oscillatory state (Fig. S4). The attractor depended on the parameters K_{ij} for each edge, while most effective regulations to determine the type of attractors were related to gene x_1, as in the four-gene model. In the five-gene model, the expression level of gene x_5 was important because the key gene x_1 is promoted via x_5.

Where oscillatory gene expression was observed, the gene expression levels in each cell were desynchronized with cell division, and the epigenetic threshold variables took different values according to cells. With the change in epigenetic variables, oscillation was attenuated or terminated for some cells, leading to differentiation.
