# Supplementary figures and images for: Pluripotency, Differentiation, and Reprogramming: A Gene Expression Dynamics Model with Epigenetic Feedback Regulation
Source: PLoS Comput Biol. 2015 Aug 26;11(8):e1004476. doi: 10.1371/journal.pcbi.1004476 (PMC4550282; doi:10.1371/journal.pcbi.1004476)

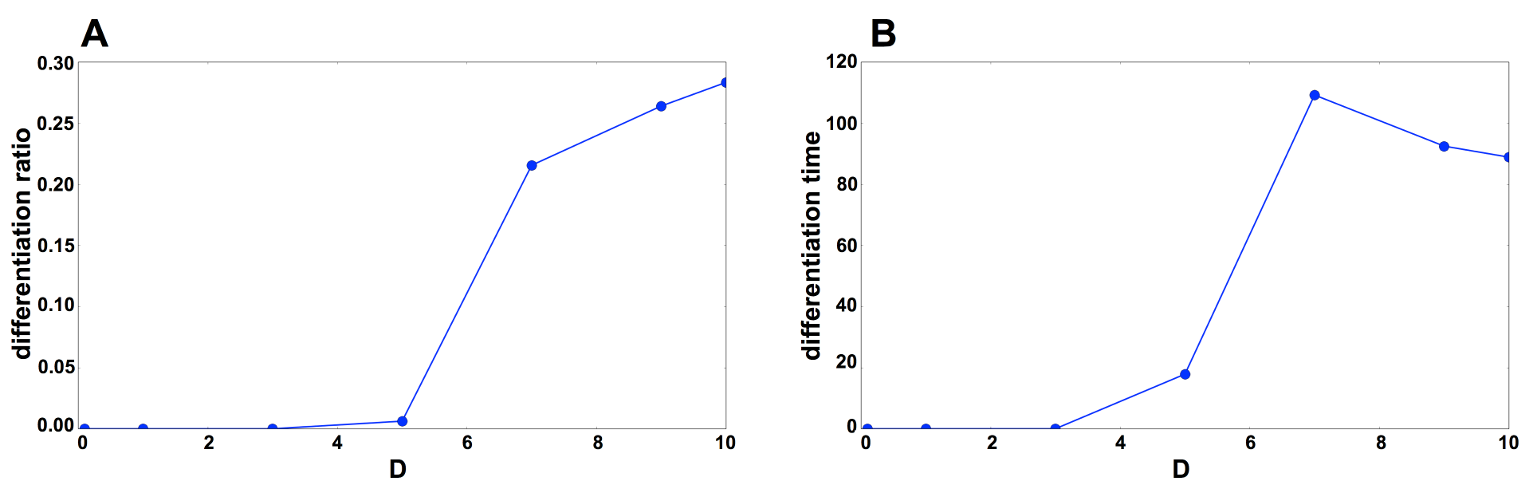

Supplement: S1 Fig — The differentiation ratio and time is plotted against the diffusion coefficient D. The simulation in the four-gene model was conducted with cell-cell interactions, and with an increase in cell numbers. From 1000 samples, we counted the number of differentiated cells (x 1 ∼ 0) for each diffusion coefficient D. A: The average of the differentiation ratio (vertical axis) was computed as the percentage of differentiated cells per simulation. Starting from a pluripotent state, the number of cells that went to a fixed point x 1 ∼ 0 increased with the diffusion coefficient D. B: The average time needed for cells to differentiate was computed and plotted as a function of D. The time to differentiation increased with the diffusion coefficient D. (TIF) [file pcbi.1004476.s002.tif]

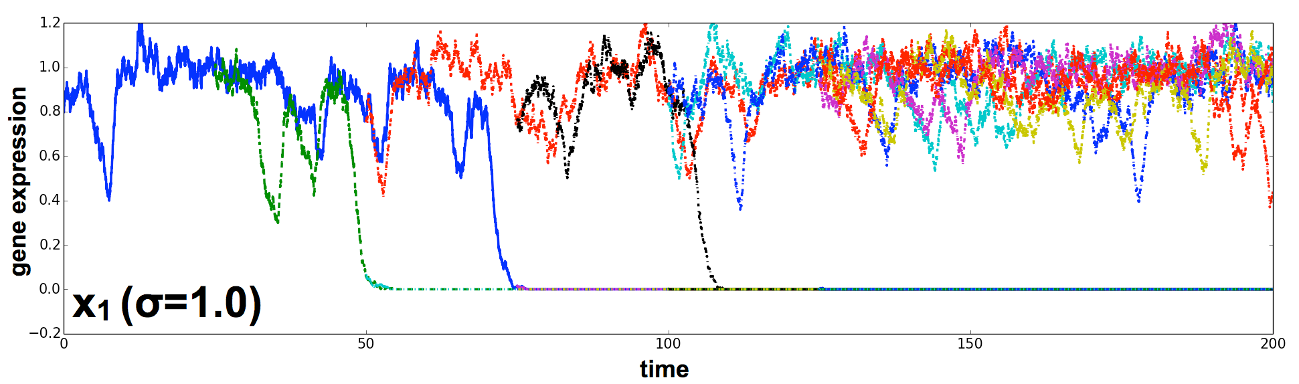

Supplement: S2 Fig — Time series of gene expression levels for x 1 (as in Fig 5). Similar conditions to those described in Fig 4 were adopted, except that a Gaussian noise term with the amplitude σ = 1.0 was included. Expression levels of cells are plotted according to color. Gene expression oscillation was irregular because of the noise. Irreversible transition from the oscillatory pluripotent to the differentiated state (x 1 ∼ 0) occurred for σ = 0.1. (TIF) [file pcbi.1004476.s003.tif]

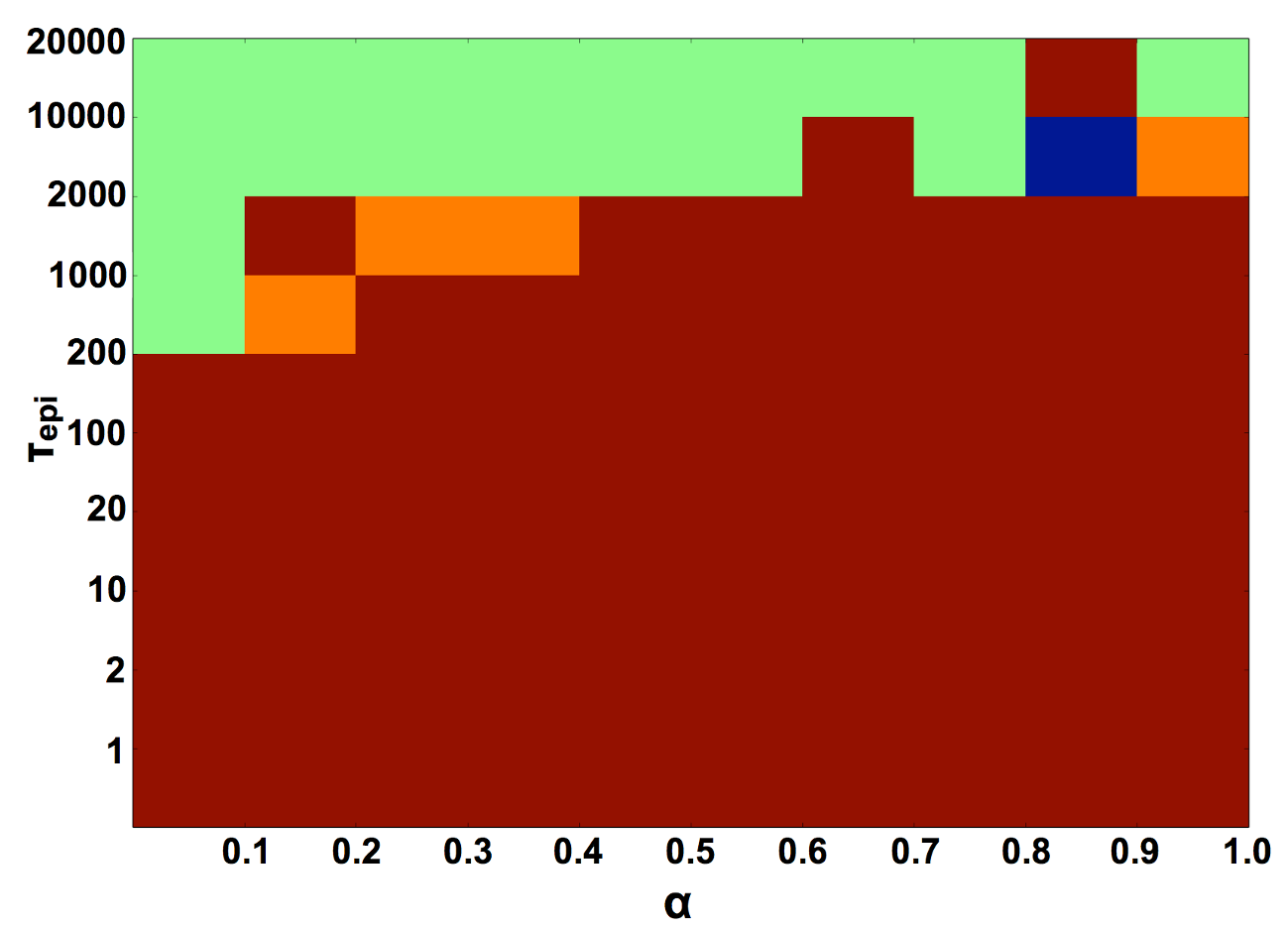

Supplement: S3 Fig — Parameters were set as in Fig 6. Brown, orange, green, and blue indicate fixed points at x 1 = 0 without differentiation, differentiation and loss of oscillation, preservation of oscillation, and the fixed-point of the differentiated state (x 1 ∼ 0) (FD), respectively. For low values of τ epi, the epigenetic fixation progressed quickly, and then cells reached the fixed-point with expressed pluripotent genes (FP). Differentiation from the oscillatory state appeared at a high value of τ epi. (TIF) [file pcbi.1004476.s004.tif]

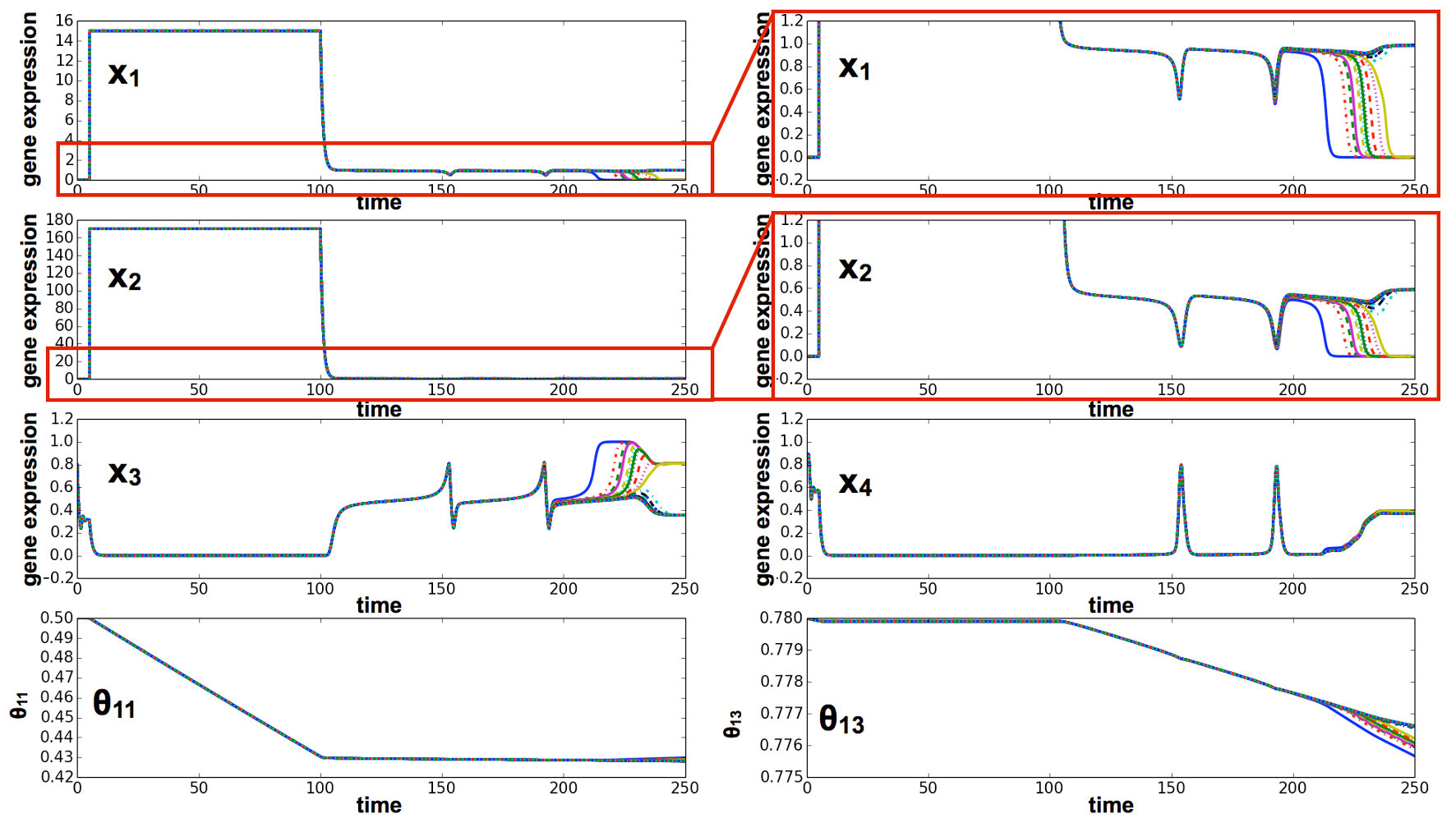

Supplement: S4 Fig — Specifically, time series of gene expression for x 1, x 2, x 3, x 4, and the epigenetic variables θ 11 and θ 13 are shown. The initial condition was as follows: all cells were in the differentiated state, where the epigenetic threshold values were set at 1.0 for the pluripotent genes Θ31, Θ21, and Θ42, and lowered for the differentiation regulators to Θ13 = 0.78, Θ34 = 0.5, Θ43 = 0.3. The value of the auto-regulator Θ11 was set at 0.50. In differentiated cells, genes x 1 and x 2 were overexpressed for a long period. The epigenetic threshold θ ij decreased with the overexpression of these genes, and the gene expression restarted oscillation. Later, a few cells differentiated again; thus, cells were reprogrammed. (TIF) [file pcbi.1004476.s005.tif]

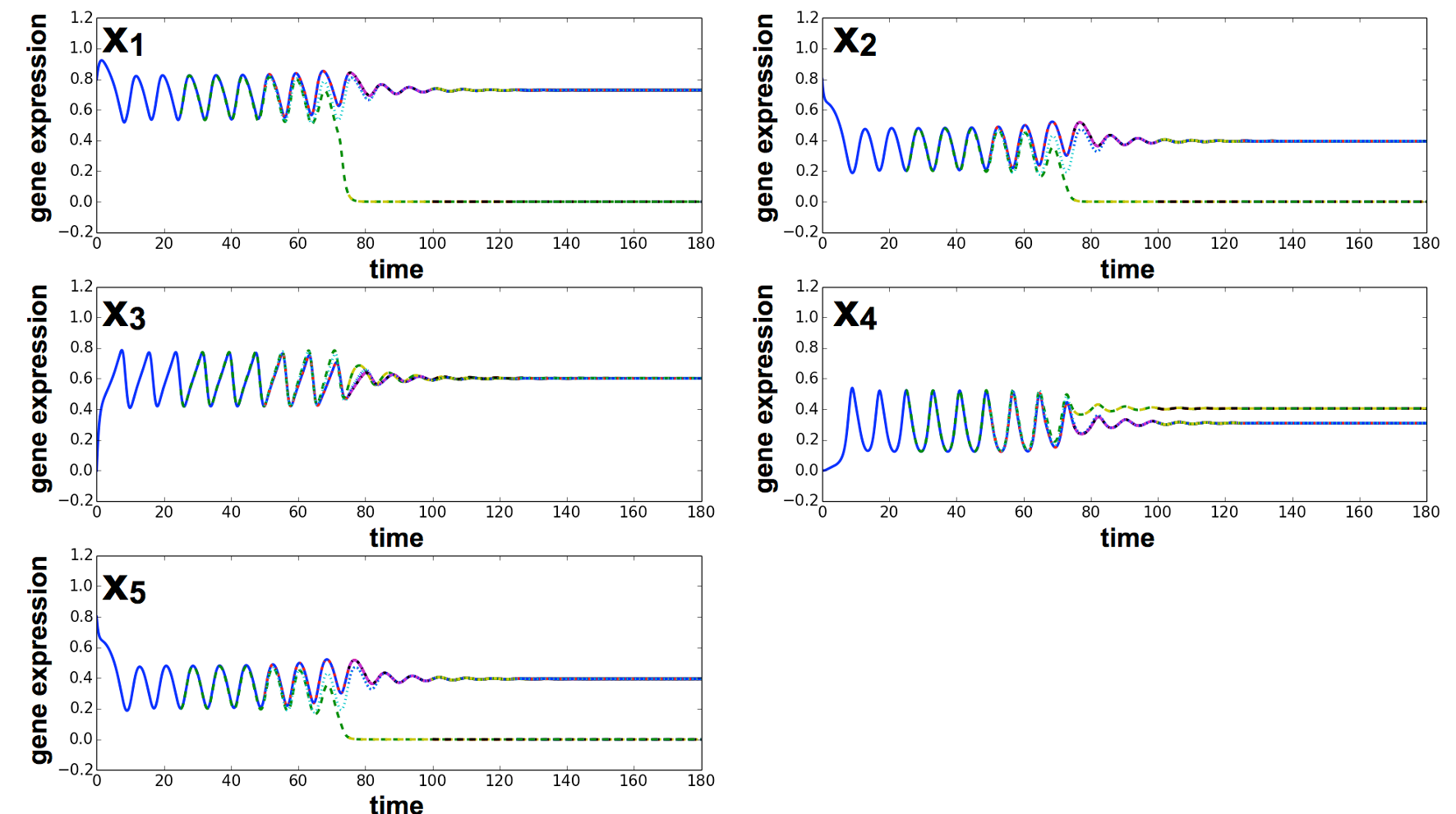

Supplement: S5 Fig — Time series of gene expression levels for x 1, x 2, x 3, x 4, and x 5. Expression levels of cells are plotted according to color, but most colors are overlaid and, therefore, difficult to discern. Here, the following parameter set was used: K 13 = 0.80, K 34 = 0.45, K 43 = 0.45, K 15 = 0.14K 31 = 0.94, K 21 = 0.81, K 51 = 0.81, and K 42 = 0.30. Gene expression levels initially showed oscillation, and then they were desynchronized. Ultimately, this model showed differentiation, as observed in the four-gene model. (TIF) [file pcbi.1004476.s006.tif]

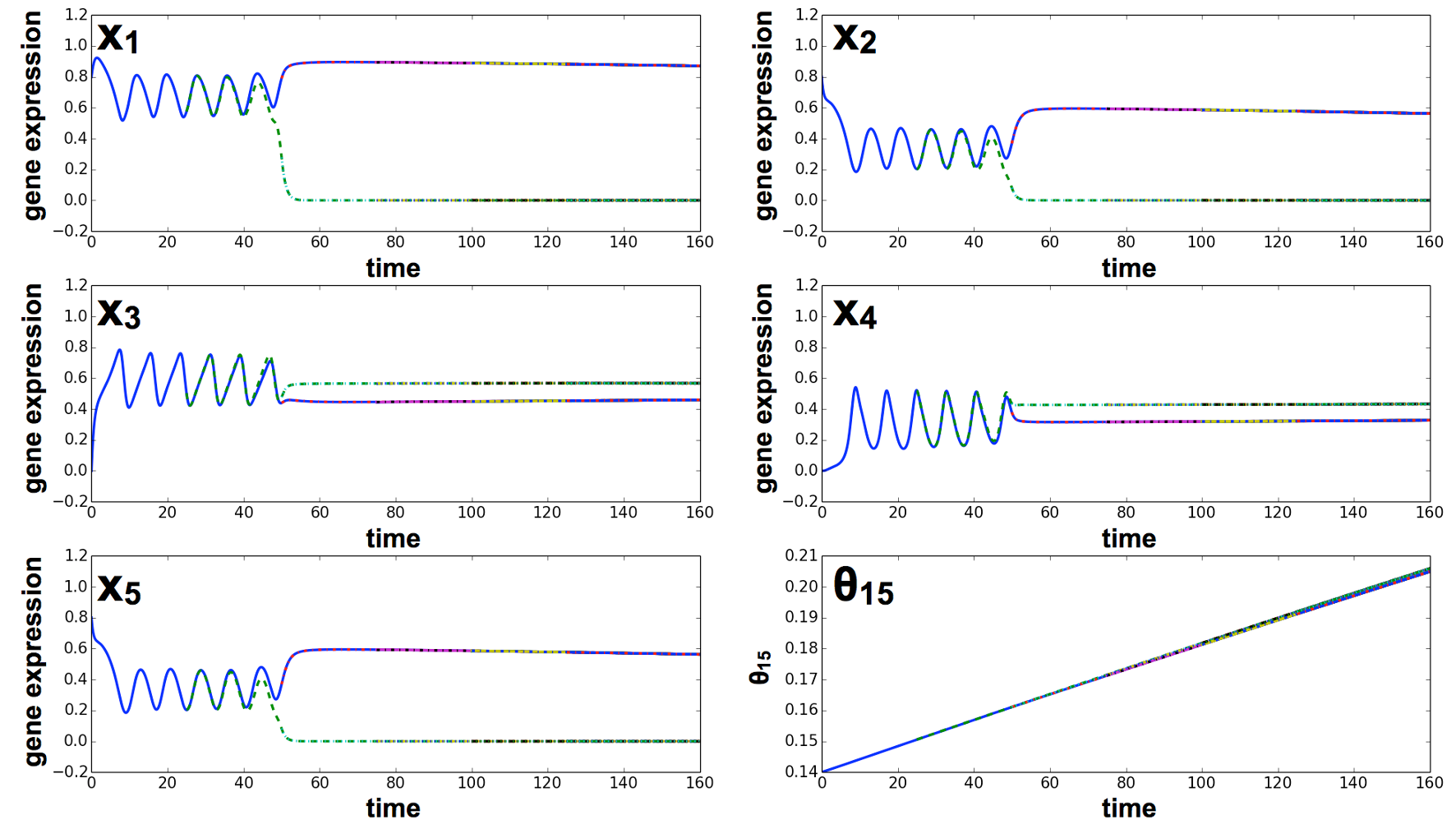

Supplement: S6 Fig — Time series of gene expression levels for x 1, x 2, x 3, x 4, x 5, and the epigenetic threshold variables θ 15(t). Here, we used parameters of Θij as follows: Θ13 = Θ34 = Θ43 = 0.65, Θ15 = Θ31 = Θ21 = Θ51 = Θ42 = 1.0. Initially, gene expression oscillated and gradually desynchronized with cell division. Ultimately, cells fell into a fixed point x 1 ∼ 1 or x 1 ∼ 0 because of epigenetic fixation. (TIF) [file pcbi.1004476.s007.tif]

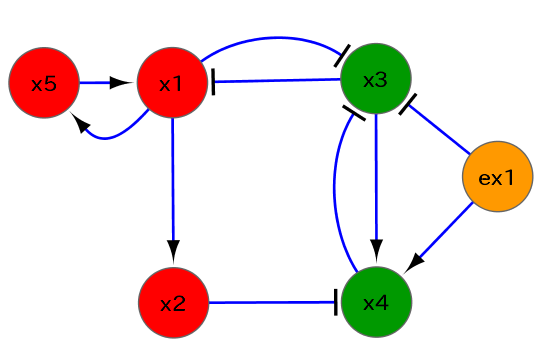

Supplement: S7 Fig — The GRN for reprogramming in the five-gene model and an external factor for reprogramming. Arrow headed and T-headed lines represent positive and negative regulation, respectively. The pluripotent genes x 1, x 2, and x 5 were overexpressed, and the external stimulus ex1 was added to inhibit x 3. Cells were reprogrammed by the induction of these factors, by which cells restarted oscillation. These inducing factors corresponded to the Yamanaka factors (Oct4, Sox2, Klf4, and Myc). (TIF) [file pcbi.1004476.s008.tif]
